# Spatial and temporal distribution of Brazilian spotted fever in an endemic area: epidemiological profile of a zoonosis in constant adaptation to anthropogenic actions

**DOI:** 10.1590/1980-549720260020

**Published:** 2026-05-08

**Authors:** Jardel Brasil, Adriano Pinter, Rodrigo Nogueira Angerami, Maria Rita Donalisio

**Affiliations:** I Health Surveillance Unit, Health Secretariat of Americana, Americana, SP, Brazil.; II Graduate Program in Public Health, Universidade Estadual de Campinas, Campinas, SP, Brazil.; III School of Veterinary Medicine and Animal Science, Universidade de São Paulo, São Paulo, SP, Brazil.

**Keywords:** Brazilian spotted fever, One health, Spatial analysis, Outbreaks

## Abstract

**Objective:**

To analyze the spatial and temporal distribution of probable infection sites (PIS) for Brazilian spotted fever (BSF) and to evaluate changes in the occurrence pattern in an endemic area in the interior of the state of São Paulo.

**Methods:**

A cross-sectional study was conducted with 40 confirmed cases of BSF (2007–2024) with identified PIS in the municipality of Americana, São Paulo. Data were obtained from the Notifiable Diseases Information System (*Sistema de Informação de Agravos de Notificação*), epidemiological investigation reports, and acarological surveys. For each georeferenced PIS, data on microclimate, altimetry, slope, Normalized Difference Vegetation Index (NDVI), and proximity to water bodies were analyzed.

**Results:**

Cases were atypically concentrated in the first half of the year, differing from the expected pattern for areas of transmission by *Amblyomma sculptum*, influenced by the outbreak recorded in 2018. PIS were concentrated near water bodies, in areas of degraded riparian forest and microclimates characterized by mild temperatures and high humidity, with low NDVI values, low altitude, and low slope. Spatial clustering was observed, along with an expansion of transmission toward urbanized areas over the study period: initially (2007–2015, nine cases) in the northern/northeastern portion of the municipality, in bordering regions, and more recently (2016–2024, 25 cases) advancing toward central areas, with the formation of large clusters.

**Conclusion:**

The findings indicate an eco-epidemiological alert scenario associated with the proximity between high densities of vectors, amplifying hosts, and human populations, reinforcing the need for intensified surveillance and targeted health education starting in the second quarter of the year.

## INTRODUCTION

Brazilian spotted fever (BSF) is a re-emerging zoonotic disease with variable clinical progression, caused by the bacterium *Rickettsia rickettsii*, and is considered the most important tick-borne zoonosis. Initially described in Brazil in 1929, in the state of São Paulo, it became a public health concern in the 1980s after a prolonged period of epidemiological silence^1^. A gradual increase in the number of cases has been observed, consistently associated with high lethality ^2-4^, despite the availability of antimicrobial therapy within the Unified Health System^5^.

Its incidence is associated with the risk of human parasitism by tick vectors of the genus *Amblyomma*. The disease shows high endemicity and sustained transmission in the metropolitan region of Campinas, São Paulo (MRC), particularly in areas within the Piracicaba, Capivari, and Jundiaí (PCJ) river basin^6^, where transmission occurs through *Amblyomma sculptum*, popularly known as the "star tick."

The significant increase in the number of BSF cases in recent decades is intrinsically associated with the growth of capybara (*Hydrochoerus hydrochaeris*) populations, the primary hosts of *A. sculptum* and amplifiers of *R. rickettsii*, in anthropogenic environments. This expansion has been particularly linked to intensive agriculture, degradation of riparian forests, urbanization, and the reduction of natural predators^7-9^.

Ecological factors influence the life cycle of reservoir vectors and hosts and, consequently, the context of BSF transmission. Changes in climate patterns and in land use and occupation have therefore become matters of concern in the context of the gradual expansion and urbanization of this zoonosis^10,11^.

Georeferencing and spatial analysis have been shown to be important tools for understanding the distribution of diseases, particularly zoonoses, within an environmental context. By integrating environmental and epidemiological data, this approach provides a more precise assessment of the distribution of vectors, reservoirs, and hosts, enabling the identification of risk areas and supporting the implementation of prevention strategies^12-14^. The objective of this study was to analyze the spatial and temporal distribution of probable infection sites (PIS), identify environmental characteristics, and examine potential changes in the pattern of occurrence of BSF cases in the municipality of Americana, an endemic area within MRC, located in the interior of the state of São Paulo.

## METHODS

A cross-sectional study with an ecological component was conducted including all confirmed cases of BSF in the municipality of Americana (237,240 inhabitants), São Paulo, between 2007 and 2024. Inclusion criteria were laboratory confirmation of the case and having the PIS determined within the municipality's territory. Data sources included Acarological Survey Reports, Epidemiological Investigation Reports, and the database of the Notifiable Diseases Information System (*Sistema de Informação de Agravos de Notificação* – SINAN), provided by the Health Surveillance Unit (*Unidade de Vigilância em Saúde* – UVISA) of the Americana Municipal Health Secretariat.

The analyses included georeferenced information obtained through the Global Positioning System (GPS) for PIS and acarological surveys, as well as data on microclimate (temperature and humidity), altimetry, slope, and the Normalized Difference Vegetation Index (NDVI), which was used to assess changes in land cover and land use, as well as to estimate biomass and vegetation vigor.

Ticks were collected and identified through acarological surveys conducted in areas at risk of transmission by the Tick Surveillance and Control Program team of UVISA in Americana. The CO_2_-baited trap technique was used, and flannel dragging was occasionally performed^15^.

The elevation and slope layers were generated using a contour line file provided by the Planning Secretariat of Americana. NDVI data were incorporated using layers derived from Landsat satellite images (Landsat 2 Level 2 Collection, 30 m resolution), provided by the United States Geological Survey^16^. Buffers of 100 and 200 meters were generated to calculate the distances between PIS and the network of water bodies.

For each geographic coordinate of PIS, annual values of NDVI, humidity, temperature (minimum and maximum), elevation, and slope were obtained. In addition, information was collected on vegetation characteristics, hydrography, the presence of primary hosts for vector ticks, and the species of ixodid ticks identified at each site.

Case density maps were generated based on a point shapefile representing the coordinates of PIS. This stage of the analysis was divided into two nine-year periods: 2007 to 2015 and 2016 to 2024, in order to assess whether BSF cases were spatially aggregated or dispersed throughout the territory and to examine potential changes in spatial distribution patterns over the historical series analyzed.

The cartographic base of the state of São Paulo, available in the electronic database of the Brazilian Institute of Geography and Statistics (*Instituto Brasileiro de Geografia e Estatística*), was used to construct the thematic maps. The maps were produced using ArcGIS^®^ version 10.8 software and projected to the SIRGAS 2000/UTM Zone 23S datum.

The study was submitted to and approved by the Research Ethics Committee of the School of Medical Sciences of Universidade Estadual de Campinas (CEP/FCM/UNICAMP), under approval No. 5.474.734.

### Data Availability Statement:

The dataset that supports the results of this study is not publicly available.

## RESULTS

**Figure 1:**
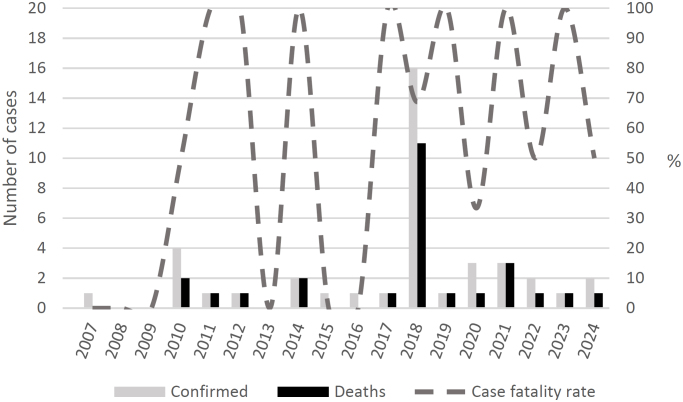
Annual distribution of autochthonous cases of Brazilian spotted fever by date of symptom onset, Americana (SP), 2007–2024.

**Figure 2:**
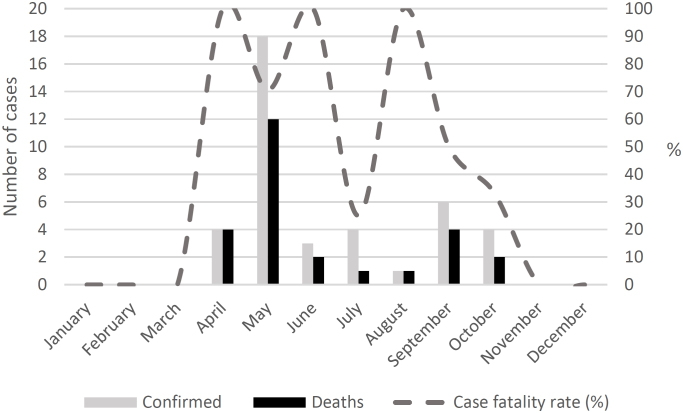
Monthly distribution of autochthonous cases of Brazilian spotted fever, by date of symptom onset, Americana (SP), 2007–2024.

Forty confirmed cases of BSF were identified in the municipality of Americana between 2007 and 2024. For six cases (15.0%), it was not possible to accurately georeference PIS within the municipal territory. The temporal distribution of cases indicates that the years 2010 (four cases) and 2018 (16 cases) presented the highest incidence (Figure 1). Figure 2 shows a concentration of confirmed cases during the first semester, with a higher frequency in May (18 cases). Variation in case fatality rates was observed throughout the study period, ranging from 0 to 100%; however, the small number of cases does not allow for a stable estimate of this indicator.

During the same period, 51,596 ticks were collected and identified in 154 acarological surveys. Of these, 17,162 (33.3%) were *Amblyomma sculptum* (12,330 larvae, 1,631 nymphs, and 3,201 adults); 3,308 (6.4%) were *Amblyomma dubitatum* (1,229 larvae, 540 nymphs, and 1,539 adults); and 31,126 (60.3%) were *Amblyomma* sp. (21,384 larvae and 9,742 nymphs). The presence of primary hosts for ticks was recorded at all georeferenced PIS, with capybaras present in 61.5% of the sites and horses occurring together with capybaras in 38.5%.

**Figure 3:**
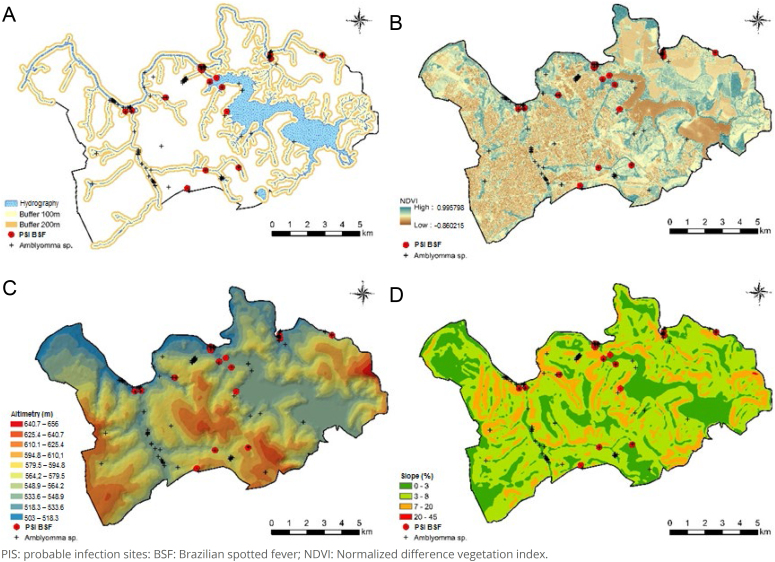
Spatial distribution of probable infection sites and ticks of the genus *Amblyomma* in the municipality of Americana (SP), 2007–2024. Buffer of 200 m (A), NDVI (B), altimetry (C), and slope (D).

**Figure 4:**
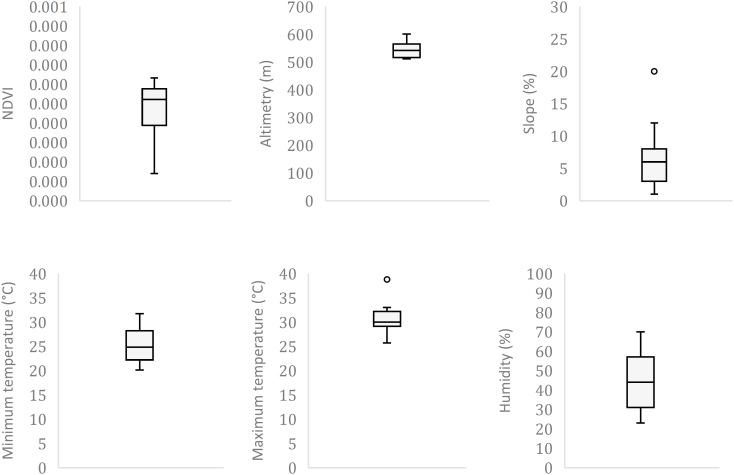
Environmental parameters of the probable infection sites for Brazilian spotted fever in Americana (SP), 2007–2024.

The spatial distribution of probable infection sites and the occurrence of ticks of the genus *Amblyomma* are presented in Figure 3. Cases were predominantly concentrated near the banks of water bodies, with 77.5% (31) located within a 100 m radius of the riverbank and 80.0% (32) within a distance of up to 200 m. These locations were characterized by areas with low NDVI values, ranging from 0.070 to 0.316 (median 0.261), low altitude — between 512 and 601 m (median 542 m) — and low slope — between 2 and 20% (median 6%). Minimum temperatures ranged from 20.1 to 31.7 °C (median 24.8 °C), maximum temperatures from 25.7 to 38.8 °C (median 30.0 °C), and average relative humidity varied between 23 and 70% (median 44%) (Figure 4).

**Figure 5:**
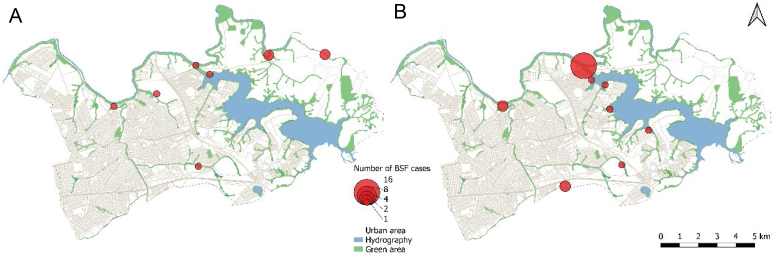
Density of cases of Brazilian spotted fever in the municipality of Americana (SP), 2007–2015 (A) and 2016–2024 (B).

The areas of case concentration for the two nine-year periods are presented in Figure 5. Between 2007 and 2015, nine cases were georeferenced (Figure 5A), representing 22.5% of the total cases during the study period. These cases were concentrated mainly in the north/northeastern portion of the municipality, with two clusters of two cases located in an environmental preservation area. Figure 5B shows the spatial distribution of the 25 georeferenced cases for the period 2016–2024, representing 62.5% of the total cases analyzed in the historical series. A cluster of 16 cases stands out, located on the northern border, along with two clusters of two cases each, on the northern and southern border regions respectively, in addition to five scattered cases distributed in more central and urbanized areas of the municipality.

## DISCUSSION

The confirmation of BSF cases was atypically concentrated in the first half of the year, differing from the pattern expected in areas where transmission occurs through *A. sculptum*, due to the disease outbreak that occurred in 2018 ^17^, considered the largest ever recorded in Brazil. In endemic areas, most cases are expected to occur during the period of greatest abundance of nymphal stages, typically between July and November^18^, as this life stage presents the highest vector competence for transmitting *Rickettsia* to humans^19^. In the municipality of Americana, however, cases were concentrated between April and June, a period in which larvae predominate in the environment.

According to Costa et al.^20^, even in endemic areas, the proportion of ticks capable of maintaining infection is very low (≤1%). This is attributed to the reduced reproductive efficiency of infected females, the low effectiveness of transovarian transmission, and the low susceptibility of *A. sculptum* to infection, in addition to the deleterious effects of *R. rickettsii* on ixodid tick populations^19^. Amplification of the infection occurs during the autumn months, when host-seeking activity begins. The few infected larvae transmit the bacterium to capybaras, leading susceptible individuals to develop sufficient rickettsemia to infect new ticks^21^. Consequently, when these ticks reach the nymphal stage, there is a substantial increase in the number of ticks capable of transmitting the pathogen to the human population.

To explain the increase in the number of cases and the occurrence of cases and outbreaks even during less favorable periods (in months with a predominance of larvae), the following stand out: the high local infestation by immature stages of the vector A. sculptum, proven through acarological surveys, and the high rate of turnover of susceptible individuals in the capybara population — possibly caused by the improved carrying capacity of the environment resulting from the return of regular rainfall after periods of extreme drought recorded in the summers of 2013/2014 and 2014/2015.^22-24^. This context likely compensated for the low vector competence of the larval stages.

A lower number of BSF case notifications was observed at the beginning of the transmission period, precisely when an increase in case incidence would be expected, as reported by other authors^4^. This finding suggests low sensitivity of the health system during the initial stages of the transmission cycle. An increase in clinical suspicion tends to occur toward the end of this period, when cases are already less frequent, which may reflect a delayed response from the health system, possibly due to reduced attention from health professionals or the difficulty of early diagnosis.

The LPIs were spatially concentrated and unevenly distributed across the territory in the northern/northeastern and southern portions of the municipality, in areas of degraded vegetation, characterized by low NDVI values, and regions of low altitude and low slope bordering water bodies, close to urban areas, coinciding with the spatial distribution of ticks of the genus Amblyomma.

The municipality of Americana presents environmental characteristics that favor the occurrence of BSF cases. Its extensive hydrographic network is particularly noteworthy, consisting mainly of the Jaguari, Atibaia, and Piracicaba rivers, the Salto Grande Reservoir, and the Quilombo streams. This environment favors the establishment of capybara populations and, consequently, areas with high infestation rates of *A. sculptum*, a vector that benefits from the abundance of primary hosts and from a microclimate characterized by mild temperatures and high humidity^25-27^. The circulation of *R. rickettsii* in the riparian forest areas of the municipality has been demonstrated through the registration of laboratory-confirmed cases, with a case fatality rate of 65.7%^28^.

Endemic areas in the municipality of Americana were characterized by high rates of tick infestation in both the environment and vertebrate hosts, as well as by the predominance of *A. sculptum*, in populations sustained essentially by capybaras, when compared to non-endemic areas, where *A. dubitatum* predominates^29^. Differences in capybara behavioral patterns have also been observed that may be associated with the increased density of *A. sculptum* in these landscapes. These include changes in habitat selection strategies^30^, a reduction in home range (2.43 times smaller), movements over shorter distances, and a tendency toward more nocturnal activity^31^, indicating the impacts of human activity on the behavior of these animals. The proximity of BSF cases to urban areas raises the possibility of the involvement of other synanthropic species as potential amplifying hosts. Serpa et al.^32^ investigated the relationship between small mammal communities in these locations and the prevalence of rickettsioses. In endemic areas, the predominance of opossums (*Didelphis albiventris*) was observed as the main hosts for ticks, predominantly infested by *A. sculptum* and presenting the highest seroprevalence titers against *R. rickettsii*. Similarly to the pattern reported by Luz et al.^29^ for capybaras, a reversal in parasitism patterns among small mammals from non-endemic areas was also observed, characterized by the predominance of *A. dubitatum* and lower infestation loads.

The change in the epidemiological profile of BSF occurrence, from a disease previously considered rural, with exposure and transmission associated with occupational activities, to an illness presenting characteristics of urban transmission associated with leisure activities, was consistent with findings reported in other published studies^3,4,33,34^. Areas of transmission, initially located in the peripheral regions of the municipality within the environmental preservation area of Americana, expanded to more central and urbanized areas of the municipality, which are characterized by high population density.

Studies investigating rare diseases, such as BSF, face methodological challenges and limitations that may compromise the robustness of analyses, particularly in the detection of more subtle patterns or in the generalization of results. Although the study area is restricted to the municipality of Americana, it is located within a region of high BSF endemicity, with a transmission scenario similar to that observed in municipalities within the PCJ hydrographic basin. Because the analysis is based on secondary data, underreporting and incomplete records may limit a more comprehensive evaluation of the cases.

The results of this study indicate an eco-epidemiological alert scenario for the transmission of BSF, characterized by a strong association between the high density of *Rickettsia* amplifying hosts and vector ticks in close proximity to human dwellings. These findings reinforce the importance of intensifying educational campaigns and targeted surveillance actions during the second quarter of the year in order to increase diagnostic sensitivity and response capacity at the most critical period of disease transmission.
