# Supplementary material for: Spatial and temporal distribution of Brazilian spotted fever in an endemic area: epidemiological profile of a zoonosis in constant adaptation to anthropogenic actions
Source: Rev Bras Epidemiol. 2026 May 8;29:e260020. doi: 10.1590/1980-549720260020 (PMC13159492; doi:10.1590/1980-549720260020)
Supplement: Translation

# Translation: Distribuição espacial e temporal da febre maculosa brasileira em área endêmica: perfil epidemiológico de uma zoonose em constante adaptação às ações antrópicas

**DOI:** 10.1590/1980-549720260020.2

**Authors:** Jardel Brasil, Adriano Pinter, Rodrigo Nogueira Angerami, Maria Rita Donalisio

**Affiliations:** I Unidade de Vigilância em Saúde, Secretaria de Saúde de Americana, Americana, SP, Brasil.; II Programa de Pós-Graduação em Saúde Coletiva, Universidade Estadual de Campinas, Campinas, SP, Brasil.; III Faculdade de Medicina Veterinária e Zootecnia, Universidade de São Paulo, São Paulo, SP, Brasil.

**Keywords:** Febre maculosa brasileira, Saúde única, Análise espacial, Surtos

## Abstract

**Objetivo:**

O objetivo deste estudo é analisar a distribuição espacial e temporal de locais prováveis de infecção (LPI) para febre maculosa brasileira (FMB) e avaliar mudanças no padrão de ocorrência em uma área endêmica do interior do estado de São Paulo.

**Métodos:**

Realizou-se estudo transversal com 40 casos confirmados de FMB (2007–2024) com LPI identificados no município de Americana/SP. Utilizaram-se dados do Sistema de Informação de Agravos de Notificação, relatórios de investigação epidemiológica e de pesquisas acarológicas. Para cada LPI georreferenciado, foram analisados dados de microclima, altimetria, declividade, *Normalized Difference Vegetation Index* (NDVI) e proximidade de corpos hídricos.

**Resultados:**

Os casos concentraram-se de forma atípica no primeiro semestre, diferente do que é esperado para áreas de transmissão por *Amblyomma sculptum*, por influência do surto registrado em 2018. Os LPI concentraram-se próximos a corpos hídricos, em áreas de mata ciliar degradada e microclima de temperaturas amenas e alta umidade, com baixo valor de NDVI, baixa altitude e declividade, apresentando agregação espacial e expansão da transmissão para áreas urbanizadas ao longo do período: inicialmente (2007–2015, nove casos) na porção norte/nordeste, em regiões limítrofes do município e, mais recentemente (2016–2024, 25 casos), avançando para áreas centrais, com formação de grandes aglomerados.

**Conclusão:**

Os achados configuram um cenário de alerta ecoepidemiológico, associado à proximidade entre alta densidade de vetores, hospedeiros amplificadores e populações humanas e reforçam a necessidade de vigilância intensificada e educação em saúde direcionada já no segundo trimestre do ano.

## INTRODUÇÃO

A febre maculosa brasileira (FMB) é uma doença reemergente, de caráter zoonótico e evolução variável, causada pela bactéria *Rickettsia rickettsii* e considerada a zoonose de maior importância transmitida por carrapatos. Inicialmente descrita no Brasil em 1929, no estado de São Paulo, tornou-se um problema de saúde pública na década de 1980, após longo período de silêncio epidemiológico^1^. Registra-se gradativo aumento no número de casos, continuamente associado a alta letalidade^2-4^, apesar da terapêutica antimicrobiana disponível no Sistema Único de Saúde^5^.

Com incidência associada ao risco de parasitismo humano por carrapatos vetores do gênero *Amblyomma*, apresenta alta endemicidade e transmissão mantida na região metropolitana de Campinas/SP (RMC), particularmente nas áreas banhadas pela bacia dos rios Piracicaba, Capivari e Jundiaí (PCJ)^6^, onde é transmitida por *Amblyomma sculptum*, popularmente conhecido como "carrapato-estrela".

O aumento significativo do número de casos de FMB nas últimas décadas está intrinsecamente associado ao aumento da população de capivaras (*Hydrochoerus hydrochaeris*), hospedeiros primários para *A. sculptum* e amplificadores de *R. rickettsii,* em ambientes antropogênicos, particularmente pela agricultura intensiva, degradação de matas ciliares, urbanização e redução de predadores^7-9^.

Fatores ecológicos exercem influência no ciclo de vida de vetores reservatórios e hospedeiros e, consequentemente, no contexto de transmissão da FMB. Mudanças nos padrões climáticos e de uso e ocupação do solo tornam-se objetos de preocupação diante de um cenário de gradativa expansão e urbanização dessa zoonose^10,11^.

O georreferenciamento e a análise espacial têm-se mostrado importantes ferramentas para compreender a distribuição de doenças, particularmente as zoonoses, no contexto ambiental. Ao combinar dados ambientais e epidemiológicos, essa abordagem oferece uma visão mais precisa da distribuição de vetores, reservatórios e hospedeiros, identifica áreas de risco e auxilia na implementação de estratégias de prevenção^12-14^. O objetivo deste trabalho é analisar a distribuição espacial e temporal de locais prováveis de infecção (LPI), identificar aspectos ambientais e eventuais mudanças no padrão de ocorrência de casos de FMB no município de Americana, área endêmica da RMC, interior do estado de São Paulo.

## MÉTODOS

Realizou-se um estudo transversal com componente ecológico com todos os casos confirmados de FMB no município de Americana (237.240 habitantes), São Paulo, entre os anos de 2007 e 2024. Na composição da amostra, utilizou-se como critério de inclusão a confirmação laboratorial do caso e ter o LPI determinado no território do município. As fontes de dados utilizadas foram os Relatórios de Pesquisas Acarológicas, Relatórios de Investigações Epidemiológicas e o banco de dados do Sistema de Informação de Agravos de Notificação (SINAN) cedidos pela Unidade de Vigilância em Saúde (UVISA) da Secretaria de Saúde de Americana.

Foram incluídas nas análises informações georrefenciadas por *Global Position System* (GPS) dos LPI e pesquisas acarológicas, além de dados sobre o microclima (temperatura e umidade), altimetria, declividade e *Normalized Difference Vegetation Index* (NDVI), utilizado para avaliar mudanças de cobertura e uso de solo, na estimação de biomassa e vigor da vegetação.

Os carrapatos foram coletados e identificados por meio de pesquisas acarológicas realizadas em áreas de risco de transmissão pela equipe do Programa de Vigilância e Controle de Carrapatos da UVISA de Americana, utilizando-se a técnica das armadilhas atrativas de CO_2_ e, eventualmente, o arrasto de flanela^15^.

As camadas de altimetria e declividade foram geradas por meio de um arquivo de curvas de nível, cedido pela Secretaria de Planejamento de Americana. Os dados sobre NDVI foram incluídos por meio de camadas elaboradas com base em imagens do satélite Landsat (Coleção Landsat 2 Nível 2, resolução de 30m), disponibilizadas pelo *United States Geological Survey* ^16^. Foram gerados *buffers* de 100 e de 200 metros para calcular as distâncias entre os LPI e a rede de corpos hídricos.

Para cada coordenada geográfica de LPI foram obtidos valores anuais de NDVI, umidade, temperatura (mínima e máxima), elevação e declividade, além de informações sobre características da vegetação, hidrografia, presença de hospedeiros primários para carrapatos vetores e espécies de ixodídeos coletados no local.

Foram elaborados mapas de densidade de casos com base no arquivo *shapefile* de pontos representado pelas coordenadas dos LPI. Esta etapa da análise foi dividida em dois períodos de nove anos: 2007 a 2015 e 2016 a 2024, com o objetivo de avaliar se os casos de FMB estariam agregados ou dispersos no território e analisar a possível ocorrência de mudança nos padrões de distribuição espacial ao longo da série histórica estudada.

A base cartográfica do estado de São Paulo, disponível no banco de dados eletrônico do Instituto Brasileiro de Geografia e Estatística, foi empregada para a construção dos mapas temáticos. Os mapas foram elaborados no *software* ArcGis^®^ versão 10.8 e reprojetados para o Datum SIRGAS 2000/UTM zona 23S.

O estudo foi submetido ao Comitê de Ética em Pesquisa da Faculdade de Ciências Médicas da Universidade Estadual de Campinas (CEP/FCM/UNICAMP) e aprovado sob parecer n° 5.474.734.

### Declaração de Disponibilidade de Dados:

O conjunto de dados que dá suporte aos resultados deste estudo não está disponível publicamente

## RESULTADOS

Foram analisados 40 casos confirmados de FMB ocorridos em Americana entre 2007 e 2024. Para seis casos (15,0%), não foi possível georreferenciar com exatidão o LPI dentro do território do município. A distribuição temporal dos casos mostra que os anos de 2010 (quatro casos) e 2018 (16 casos) se destacam por apresentarem maior incidência (Figura 1). A Figura 2 aponta concentração de casos confirmados no primeiro semestre, com maior relevância no mês de maio (18 casos), e observa-se variação nas taxas de letalidade no período, de 0 a 100%, embora o pequeno número de casos não permita uma estimativa estável do indicador.

**Figura 1:** Distribuição anual dos casos autóctones de febre maculosa brasileira, por data de início de sintomas, Americana (SP), 2007–2024.

**Figura 2:** Distribuição mensal dos casos autóctones de febre maculosa brasileira, por data de início de sintomas, Americana (SP), 2007–2024.

No mesmo período foram coletados e identificados 51.596 carrapatos em 154 pesquisas acarológicas, sendo 17.162 (33,3%) *Amblyomma sculptum* (12.330 larvas, 1.631 ninfas e 3.201 adultos), 3.308 (6,4%) *Amblyomma dubitatum* (1.229 larvas, 540 ninfas e 1.539 adultos) e 31.126 (60,3%) *Amblyomma* sp. (21.384 larvas e 9.742 ninfas). A presença de hospedeiros primários para carrapatos foi registrada para todos os LPI georreferenciados, em 61,5% com registro de capivaras e em 38,5% com ocorrência de cavalos em conjunto com capivaras.

A distribuição espacial dos locais prováveis de infecção e de ocorrência de carrapatos do gênero *Amblyomma* é apresentada na Figura 3. Observa-se que os casos se concentraram próximo às margens de corpos hídricos, com 77,5% (31) localizados em um raio de 100 m da margem e 80,0% (32) na faixa de até 200 m, em áreas de baixo NDVI, com valores na faixa de 0,070 a 0,316 (mediana 0,261), baixa altitude — entre 512 e 601 metros (mediana 542 m) — e baixa declividade — entre 2 e 20% (mediana 6%), com temperaturas mínimas que variaram de 20,1 a 31,7°C (mediana 24,8°C), máximas de 25,7 a 38,8°C (mediana 30,0°C) e umidade relativa média entre 23 e 70% (mediana 44%) (Figura 4).

**Figura 3:** Distribuição espacial dos locais prováveis de infecção e carrapatos do gênero *Amblyomma* no município de Americana (SP), 2007–2024. *Buffer* de 200 m (A), NDVI (B), altimetria (C) e declividade (D).

**Figura 4:** Parâmetros ambientais dos locais prováveis de infecção para febre maculosa brasileira em Americana (SP), 2007–2024.

As áreas de concentração de casos para os dois períodos de nove anos são mostradas na Figura 5. Entre 2007 e 2015 foram georreferenciados nove casos (Figura 5A), 22,5% do total para o período estudado, concentrados majoritariamente na porção norte/nordeste do município, destacando-se dois aglomerados de dois casos em área de preservação ambiental. A Figura 5B apresenta a distribuição espacial dos 25 casos georreferenciados para o período de 2016–2024, representando 62,5% do total dos casos analisados na série histórica, com destaque para um aglomerado de 16 casos situado no limite norte e dois aglomerados de dois casos, nas regiões limítrofes norte e sul respectivamente, além de cinco casos esparsos distribuídos em áreas mais centrais e urbanizadas do município.

**Figura 5:** Densidade de casos de febre maculosa brasileira no município de Americana (SP), 2007–2015 (A) e 2016–2024 (B).

## DISCUSSÃO

A confirmação dos casos de FMB concentrou-se de forma atípica no primeiro semestre, diferente do que é esperado para áreas de transmissão por *A. sculptum*, em decorrência do surto da doença ocorrido em 2018^17^, considerado o maior já registrado no Brasil. Espera-se que a maioria dos casos se concentre na época de maior abundância dos estágios ninfais, entre os meses de julho a novembro^18^, por ser a fase de vida com maior competência vetorial para transmitir a *Rickettsia* ao ser humano^19^. Em Americana, os casos concentraram-se entre os meses de abril e junho, período em que as larvas predominam no ambiente.

Segundo Costa et al.^20^, mesmo em áreas endêmicas, a porcentagem de carrapatos que conseguem manter a infecção é muito baixa (≤1%) em razão da menor eficiência reprodutiva das fêmeas infectadas, da baixa efetividade da transmissão transovariana e da baixa susceptibilidade de *A. sculptum* à infecção, além do efeito deletério demonstrado por *R. rickettsii* na população de ixodídeos^19^. A amplificação acontece nos meses de outono, quando se inicia a busca por hospedeiros. As poucas larvas infectadas transmitem a bactéria para a capivara, levando os indivíduos suscetíveis a desenvolverem rickettsemia suficiente para infectar novos carrapatos^21^. Assim, quando chegam à fase de ninfa, há um aumento considerável no número de carrapatos aptos a infectar a população humana.

Para explicar o aumento no número de casos e a ocorrência de casos e surto mesmo em período menos propício (em meses com predomínio de larvas), destacam-se a grande infestação local por fases imaturas do vetor *A. sculptum*, comprovada por meio de pesquisa acarológica, e alta taxa de renovação de indivíduos suscetíveis na população de capivaras — provocada, possivelmente, pela melhora da capacidade de suporte do ambiente resultante do retorno das chuvas regulares após períodos de seca extrema registrados nos verões de 2013/2014 e 2014/2015^22-24^. Esse contexto provavelmente compensou a baixa competência vetorial dos estágios larvais.

Observou-se que a notificação dos casos de FMB foi menor no início do período de transmissão, justamente quando há tendência de aumento da incidência de casos, como registrado por outros autores^4^, sugerindo baixa sensibilidade do sistema de saúde nos estágios iniciais do ciclo. O aumento da suspeição ocorre no final desse período, quando os casos já são mais escassos, o que pode refletir uma resposta tardia do sistema, possivelmente em razão da menor atenção dos profissionais de saúde ou da dificuldade de diagnóstico precoce.

Os LPI apresentaram-se espacialmente concentrados e desigualmente distribuídos pelo território nas porções norte/nordeste e sul do município, em áreas de vegetação degradada, caracterizada por baixos valores de NDVI, e regiões de baixa altitude e declividade marginal a corpos hídricos, próximas de área urbana, coincidindo com a distribuição espacial de carrapatos do gênero *Amblyomma.*

Americana apresenta características ambientais que favorecem a ocorrência de casos de FMB. Destacam-se sua exuberante hidrografia, composta principalmente pelos rios Jaguari, Atibaia, Piracicaba, Represa do Salto Grande e Ribeirão Quilombo. Essa ambiência propicia o estabelecimento de populações de capivaras e, consequentemente, áreas com altas taxas de infestação por *A. sculptum*, vetor beneficiado pela abundância de hospedeiros primários e pelo microclima de temperaturas amenas e umidade elevada^25-27^. A circulação de *R. rickettsii* nas áreas de mata ciliar do município foi comprovada por meio do registro de casos confirmados laboratorialmente, com letalidade de 65,7%^28^.

As áreas endêmicas em Americana foram caracterizadas pelas altas taxas de infestação por carrapatos, tanto no ambiente quanto nos hospedeiros vertebrados, e pela predominância de *A. sculptum,* em populações sustentadas essencialmente por capivaras, quando comparadas a áreas não endêmicas, onde se observa o predomínio de *A. dubitatum* ^29^. Também foram verificadas diferenças nos padrões de comportamento das capivaras que podem estar relacionadas ao aumento da densidade de *A. sculptum* nessas paisagens, tais como mudanças nas estratégias de seleção de hábitats^30^, redução na área de vida (2,43 vezes menor), movimentação por menores distâncias e tendência a hábitos mais noturnos^31^, indicando os impactos da ação antrópica no comportamento desses animais.

A proximidade dos casos de FMB a áreas urbanas levanta a possibilidade do envolvimento de outras espécies sinantrópicas como potenciais hospedeiros amplificadores. Serpa et al.^32^ estudaram a relação das comunidades de pequenos mamíferos presentes nessas localidades com a prevalência de rickettsioses. Observaram nas áreas endêmicas a predominância de gambás (*Didelphis albiventris*) como principais hospedeiros para carrapatos, infestados majoritariamente por *A. sculptum* e apresentando os maiores títulos de soroprevalência contra *R. rickettsii.* Assim como relatado por Luz et al.^29^ para as capivaras, também verificaram uma inversão do parasitismo nos pequenos mamíferos de áreas não endêmicas, com dominância de *A. dubitatum* e em menores cargas.

A mudança no perfil epidemiológico de ocorrência da FMB, anteriormente considerada uma doença do meio rural com exposição e transmissão relacionada à atividade laboral, para uma enfermidade com características de transmissão urbana associada a atividades de lazer, apresentou-se em conformidade com outros estudos já publicados^3,4,33,34^. As áreas de transmissão, inicialmente localizadas nas regiões limítrofes da cidade, dentro da área de preservação ambiental de Americana, expandiram-se para regiões centrais e urbanizadas do município, com alta densidade populacional.

Estudos que investigam doenças raras, como a FMB, enfrentam desafios metodológicos e limitações que podem comprometer a robustez das análises, especialmente na detecção de padrões mais sutis ou na generalização dos resultados. Embora a área de estudo tenha abrangência limitada ao município de Americana, ela pertence à região de alta endemicidade de FMB, em cenário de transmissão semelhante ao dos municípios da bacia hidrográfica do PCJ. Por se tratar de análise de dados secundários, a subnotificação e a falta de completude dos registros podem prejudicar uma análise mais abrangente dos casos.

Os resultados encontrados neste estudo configuram um cenário de alerta ecoepidemiológico para a transmissão de FMB, estabelecido pela forte associação de alta densidade de hospedeiros amplificadores de *Rickettsia* e carrapatos vetores em proximidade com habitações humanas, e reforçam a importância de intensificar campanhas educativas e ações de vigilância direcionadas já no segundo trimestre do ano, de modo a aumentar a sensibilidade diagnóstica e a capacidade de resposta no momento mais crítico da transmissão da doença.
